# Special congenital dacryocystocele

**DOI:** 10.12669/pjms.39.2.6819

**Published:** 2023

**Authors:** Li-po Han, Feng-xian Wang, Cheng-yue Zhang

**Affiliations:** 1 Li-po Han, Department of Ophthalmology, Baoding Chilren’s Hospital, Hebei Baoding 071000, China; 2 Feng-xian Wang, Department of Ophthalmology, Baoding Chilren’s Hospital, Hebei Baoding 071000, China; 3 Cheng-yue Zhang, Department of Ophthalmology, Beijing Chilren’s Hospital, Beijing 010000, China

**Keywords:** Congenital dacryocystocele, Nasolacrimal obstruction

## Abstract

Congenital Dacryocystocele is a rare disease of the eye and nose, which originates from congenital obstruction of lacrimal duct system, but accounts for a low proportion in congenital obstruction of lacrimal duct system. We present a case of congenital dacryocystocele to analyze the clinical features and to explore the clinical treatment effect of special congenital dacryocyst protrusion.

## INTRODUCTION

Congenital dacryocystocele is a rare case in Ophthalmology.^1^ The typical congenital lacrimal sac protrusion is the first symptom to discover the blue-purple mass in the lacrimal sac area of children. Some cases can be detected by ultrasound during the embryo period.^2^,^3^ Davies R observed that 91% of the lacrimal sac protruded to one side.^4^ Twenty-three per cent of patients require surgical intervention.

## CASE PRESENTATION

**Fig.1:**
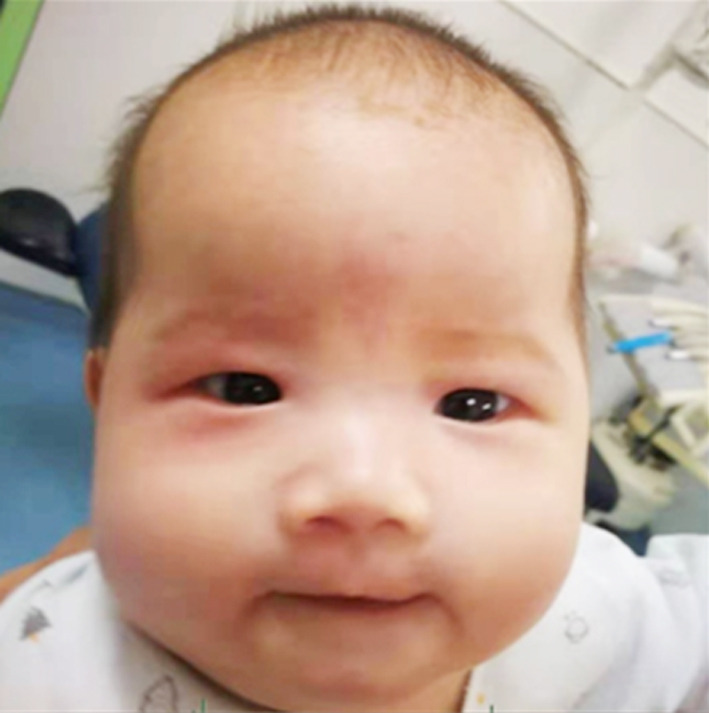


**Fig.2:**
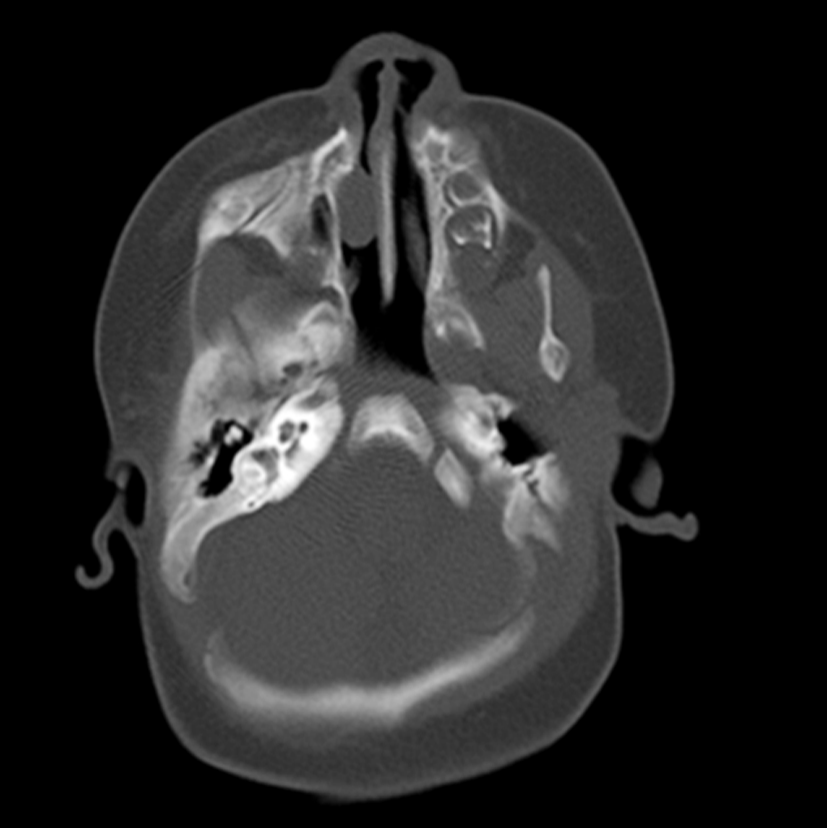


**Fig.3:**
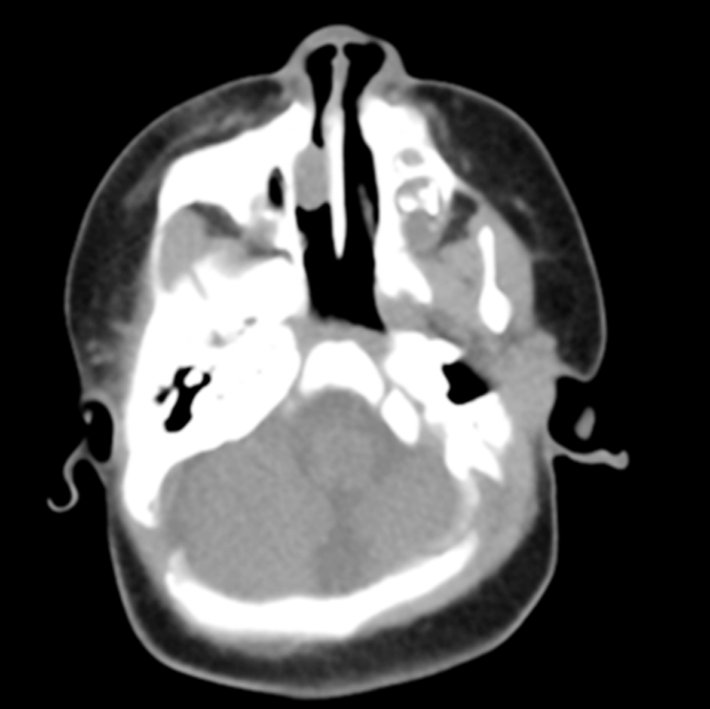


**Fig.4:**
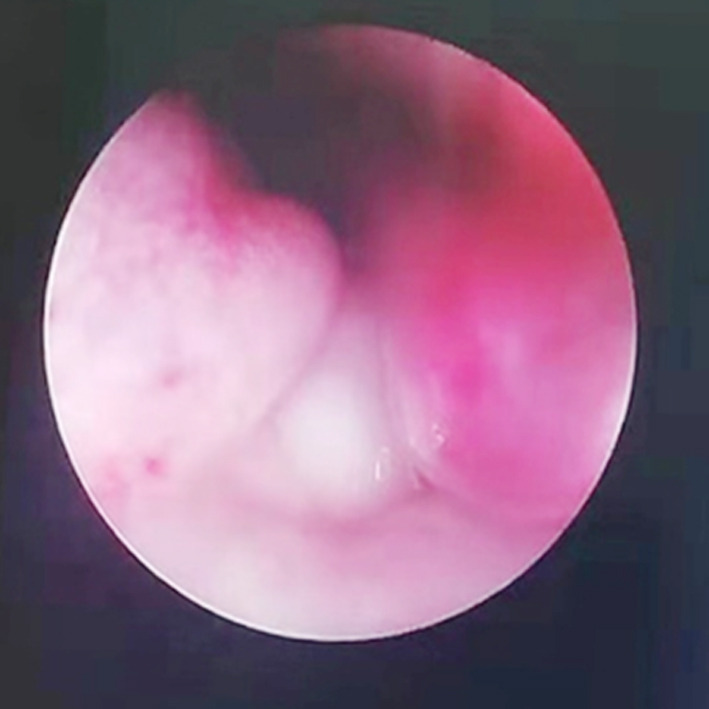


**Fig.5:**
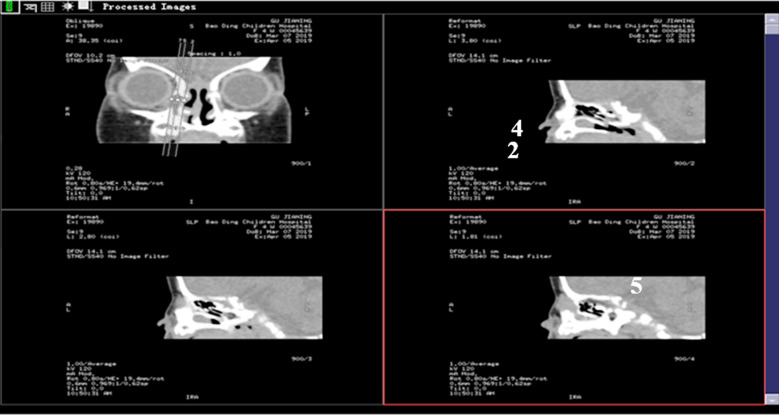


A girl 81 days after the birth, presented with the right eye tears (Fig.1). She underwent right lacrimal duct exploration in another hospital, but the treatment failed. Physical examination showed that tears flowed from the right eye, no obvious redness and swelling in the right eye, and tears oozed pus in the dacryocyst area when squeezed. The result of right tear duct flushing was found to be full reflux. CT examination and three-dimensional reconstruction of nasolacrimal duct revealed a cystic mass at the nasosacral orificium of the right lower nasal duct (Fig.2, 3 and 5). Nasal endoscopy (Fig.4): cystic masses can be seen in the lower nasal canal and diagnosed as “congenital dacryocystocele”. We have taken permission to use the photograph in the write up.

### Ethical approval:

The study was approved by the Institutional Ethics Committee of Baoding Children’s Hospital (No.: 202182; date: November 19, 2021), and written informed consent was obtained from all participants.

## DISCUSSION

Congenital dacryocystocele is a relatively common disease among neonates and infants. The frequency is reported from 1.25% to 12.5% according to authors.^5^ Congenital dacryocystocele usually appears in the first week or month of life and is caused by distal obstruction of the nasolacrimal system and functional proximal obstruction of the junction between the nasolacrimal tubules and the lacrimal sac. The result is dilation of the lacrimal sac below the medial canthal tendon, which may appear as a mass. A combination of dissolved mesoderm, mucus, amniotic fluid, tear fluid, and bacterial colonization may contribute to the swelling of the lacrimal system within the lacrimal sac.^6^,^7^

It is important to distinguish dacryocyst cysts from other diseases. Blue cystic masses without associated symptoms are often difficult to distinguish from hemangiomas. Dacryocyst cysts can be easily diagnosed by ultrasound and confirmed by probing the lacrimal duct. Ultrasound is a simple, noninvasive procedure that can reliably distinguish dacryocyst cysts from other pathologies.^8^ Further understanding of this disease can avoid premature termination of pregnancy due to mistaken fetal malformation, therefore, great attention should be paid to it.

The management of a congenital dacryocystocele is conservative, and it includes gentle massage on the lacrimal sac, which can facilitate decompression and drainage of the contents into the nose. Antibiotic drops may also be used prophylactically before infection occurs.^9^ Probing and irrigation of nasolacrimal system has been shown to effectively ameliorate the obstruction. Significant controversy exists as to the preferred management and timing of intervention for infants with congenital dacryocystoceles. After confirming the diagnosis by lacrimal excretion test and radiological examination, the outcome of appropriate surgical treatment should be successful.^10^-^12^

In this case, we found that the patient eventually underwent the “right eye and nose endoscopy” under the nasal endoscope. The symptoms of tears disappeared the next day after the operation. The lacrimal canal was washed smoothly. After three months of diagnosis, the child’s right eye had no symptoms of tears, lacrimal canal flushing. This case reminds us that congenital lacrimal sac protrusions do not necessarily have the “blue-purple swelling of the canthus” as the first symptom. In children with congenital lacrimal canal obstruction in the lacrimal canal, lacrimal CT and endoscopic examination should be performed, except where there is an atypical congenital dacryocystocele possibility. In the case of congenital dacryocystocele, which is mainly represented by nasal cyst, the surgical effect of nasal lacrimal vesicle is satisfactory.

## CONCLUSION

Congenital dacryocyst protrusion combined with infection can be complicated with cellulitis, septicemia, meningitis, brain abscess^13^, larger cyst in nasal canal can still have respiratory distress, so it should not be ignored. Surgical intervention should be done as soon as possible. The operation method includes: under the nasal endoscope the nasal cavity cyst ruptures the cyst. Early operation can not only achieve satisfactory therapeutic effect, but also avoid serious complications caused by repeated infection which endanger the life of the child.

### Authors’ contributions:

**LH:** Designed this study, prepared this manuscript, are responsible and accountable for the accuracy and integrity of the work.

**CZ:** Collected and analyzed clinical data.

**FW:** Data analysis, significantly revised this manuscript.

## References

[1] Zhao NW, Chan DK (2019). Awake bedside nasal endoscopy for primary management of neonatal dacryocystoceles with intranasal cysts. Int J Pediatr Otorhinolaryngol.

[2] Singh S, Ali MJ (2019). Congenital Dacryocystocele: A Major Review. Ophthalmic Plast Reconstr Surg.

[3] Miranda-Rivas A, Villegas VM, Nieves-Melendez JR, De La Vega A (2018). Congenital dacryocystocele: sonographic evaluation of 11 cases. J AAPOS.

[4] Davies R, Watkins WJ, Kotecha S, Watts P (2018). The presentation, clinical features, complications, and treatment of congenital dacryocystocele. Eye (Lond).

[5] Noda S, Hayasaka S, Setogawa T (1991). Congenital nasolacrimal duct obstruction in Japanese infants: its incidence and treatment with massage. J Pediatr Ophthalmol Strabismus.

[6] Wong RK, VanderVeen DK (2008). Presentation and management of congenital dacryocystocele. Pediatrics.

[7] Ha YJ, Choi HY, Myung KB, Choi YW (2010). A case of congenital dacryocystocele. Ann Dermatol.

[8] Cruciat G, Florian A, Cotutiu P, Nemeti G, Nicoara S (2020). Congenital dacryocystocele diagnosed by antenatal ultrasonography with spontaneous resolution. Arq Bras Oftalmol.

[9] Hitter A, Lamblin E, Morand B, Bertolo A, Atallah I, Righini CA (2016). Congenital dacryocystocele: Surgical treatment or routine follow-up?. Rev Stomatol Chir Maxillofac Chir Orale.

[10] Memon MN, Siddiqui SN, Arshad M, Altaf S (2012). Nasolacrimal duct obstruction in children: outcome of primary intubation. J Pak Med Assoc.

[11] Bozan N, Sakin YF, Kundi P, Ari M, Bozkus F (2017). A huge Thornwaldt's cyst causing hearing loss in an adult patient. J Pak Med Assoc.

[12] Akpolat C, Sendul SY, Unal ET, Karatas E, Ucgul Atilgan C, Demir M (2021). Outcomes of lacrimal probing surgery as the first option in the treatment of congenital dacryocystocele. Ther Adv Ophthalmol.

[13] Leonard DS, O'Keefe M, Rowley H, Hughes JP (2008). Neonatal respiratory distress secondary to bilateral intranasal dacryocystocoeles. Int J Pediatr Otorhinolaryngol.

